# Angina pectoris? Fake news: a case report of infective endocarditis with giant aortic root abscess detected by cardiac magnetic resonance imaging

**DOI:** 10.1093/ehjcr/ytae297

**Published:** 2024-06-11

**Authors:** Stephanie Wissel, Maria Drayß, Martin Christa, Rainer G Leyh, Stefan Frantz

**Affiliations:** Department of Internal Medicine I, University Hospital Würzburg, Oberdürrbacherstraße 6, Würzburg, 97080, Germany; Department of Internal Medicine I, University Hospital Würzburg, Oberdürrbacherstraße 6, Würzburg, 97080, Germany; Department of Internal Medicine I, University Hospital Würzburg, Oberdürrbacherstraße 6, Würzburg, 97080, Germany; Department of Thoracic and Cardiovascular Surgery, University Hospital Würzburg, Würzburg, Germany; Department of Internal Medicine I, University Hospital Würzburg, Oberdürrbacherstraße 6, Würzburg, 97080, Germany

**Keywords:** Aortic root abscess, Infective endocarditis, Echocardiography, Cardiac CT, Cardiac MR, Acute coronary syndrome, NSTEMI

## Abstract

**Background:**

Infective endocarditis (IE) is a rare disease associated with high mortality rates. Clinical presentation is highly variable with a time interval between first onset of symptoms and diagnosis > 1 month in 25% of patients. We present a case of aortic valve endocarditis with aortic root abscess (ARA) with chest pain and ischaemic changes on the electrocardiogram (ECG).

**Case summary:**

A 59-year-old Caucasian male with a known bicuspid aortic valve presented at our emergency department with a 2-week history of malaise, subfebrile temperatures, and chest pain episodes. The ECG exhibited ischaemic changes, and laboratory workup showed elevated inflammatory markers and troponin levels. Coronary angiography revealed a one-vessel coronary artery disease with a borderline significant stenosis of the left circumflex artery. Cardiac magnetic resonance imaging showed a large aortic valve vegetation with an ARA expanding intramyocardially, which was not seen on bedside echocardiography. The patient was set on intravenous (i.v.) antibiotics and urgently referred for surgery. The patient received surgical aortic root and valve replacements, reconstruction of the anterior mitral leaflet, and a venous bypass. After successful surgical management followed by 6 weeks of i.v. antibiotics, the patient completely recovered.

**Discussion:**

Diagnosing IE in atypical cases, such as those with ischaemic ECG changes, remains challenging. Infective endocarditis should be considered as an early differential diagnosis in individuals with prosthetic or native valve disease. Infective endocarditis poses a significant risk for perivalvular and ARA formation with high mortality. Aortic root abscess may present with unspecific symptoms or unusual ECG changes and might be missed in standard transthoracic echocardiography in up to 30% of cases. Multimodal imaging can help in establishing a prompt and accurate diagnosis, aid in timely treatment and mitigating the risk of complications of IE.

**Learning points**

- Multimodal imaging should be used more frequently in patients with predisposing risk factors (prosthetic valves, native valve disease, and cardiac implants) for infective endocarditis to ensure timely diagnosis and management of infective endocarditis.
- Computed tomography of the coronary arteries is a useful tool for non-invasive diagnosis of coronary artery disease and detection of valvular and paravalvular vegetations or abscess formations, while cardiac magnetic resonance imaging is well suited for detection of embolic or abscess formations.

## Introduction

A perivalvular abscess may arise as a severe complication of infective endocarditis (IE), affecting heart valve and surrounding tissues in up to 30% of native valve IE cases. Perivalvular complications are more common in aortic valve IE and patients with bicuspid aortic valves.^1^ This case report illustrates a patient presenting with chest pain, diagnosed with an aortic root abscess (ARA) through cardiac magnetic resonance imaging (CMR), demonstrating the diverse clinical presentations of perivalvular abscesses and highlighting the benefits of multimodality imaging, particularly following the updated ESC Endocarditis guideline in 2023.^1^

## Summary Figure

Figure for abbreviations used in timeline figure

**Table:** 

**Initial presentation**	The patient presented with a 2-week history of malaise, subfebrile temperatures, and intermittent chest pain episodes. Clinical examination was unremarkable. Troponin and inflammatory markers increased. Coronary angiography showed a borderline stenosis at the proximal ramus circumflexus.
**Day 2**	Transthoracic echocardiography (TTE) showed a moderately stenotic bicuspid aortic valve with mild aortic insufficiency. Aortic root was not dilated.
**Day 3**	Blood cultures turned positive for coagulase-negative gram-positive cocci. Intravenous (i.v.) antibiotics were started and a CMR revealed large vegetations at the aortic valve and an extensive subvalvular aortic root abscess, expanding intramyocardially. The multidisciplinary team was involved, and the patient was referred for urgent surgical intervention.
**Day 4**	Final result of initial blood cultures confirmed growth of *Staphylococcus epidermidis* sensitive to flucloxacillin in all three pairs of blood cultures. Antibiotics were changed to i.v. flucloxacillin 2 g every 4 h. Transoesophageal echocardiography (TEE) confirmed the aortic root abscess and bicuspid aortic valve endocarditis with mild regurgitation and normal left ventricular ejection fraction. Preoperative computed tomography (CT) scan for precise surgical planning was performed and demonstrated a ‘road of pus’ formed by the paravalvular abscess, expanding from the anterior cusp of the bicuspid valve to the anterior basal ventricular myocardium.
**Day 5**	Surgical debridement of the abscess, replacement of the aortic valve and aortic root by a homograft, and replacement of the anterior leaflet of the mitral valve were performed. The pulmonary artery was reconstructed and a venous bypass was inserted.
**2 and 4 weeks post-surgery**	Transthoracic echocardiography repeatedly showed a well-seated aortic homograft and prosthetic tissue valve. Peak gradient of 8 mmHg. Left ventricular ejection fraction estimated at 60%.
**Day 42**	Four weeks of i.v. antibiotic therapy after first negative blood culture was completed and switched to oral levofloxacin and rifampicin for two more weeks. The patient was discharged from hospital to a rehabilitation facility.

### Case presentation

**Figure 1:**
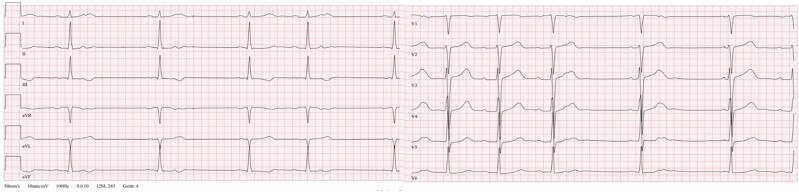
Electrocardiogram at admission showing T-wave inversions in III, aVR, and aVF, broadened P-waves, and varying lengths of PR intervals associated with aortic root abscess and close proximity of the atrioventricular node to the aortic valve.

A 59-year-old Caucasian male with a known bicuspid aortic valve presented at our emergency department with a 2-week history of malaise, subfebrile temperatures, and chest pain episodes. The patient was a non-smoker with no history of drug abuse, no history of coronary artery disease (CAD), and no recent operative procedure or previous heart surgery. His last dental intervention was a dental cleansing procedure 3 months prior to onset of symptoms. The clinical examination at presentation was unremarkable with no signs of infection and stable vital signs. The electrocardiogram (ECG) showed T-wave inversions in III, aVR, and aVF and varying lengths of PR intervals (*Figure 1*). Blood tests revealed a significantly increased high-sensitivity troponin (hsT) value (369 pg/mL) and elevated inflammatory markers (C-reactive protein 13.8 mg/dL, white blood cell (WBC) count 11.5 * 100/μL, thrombocytes 485 * 1000/μL) but otherwise normal renal, liver, and thyroid functions. Urinalysis showed no abnormalities. The polymerase chain reaction panel for respiratory viruses remained negative. Chest X-ray showed no pulmonary infiltrates, pleural effusion, or pneumothorax. Bedside echocardiography, which suffered from a limited acoustic window, showed a mildly reduced ejection fraction with global hypokinesis and a calcified, moderately stenotic bicuspid aortic valve.

**Figure 2:**
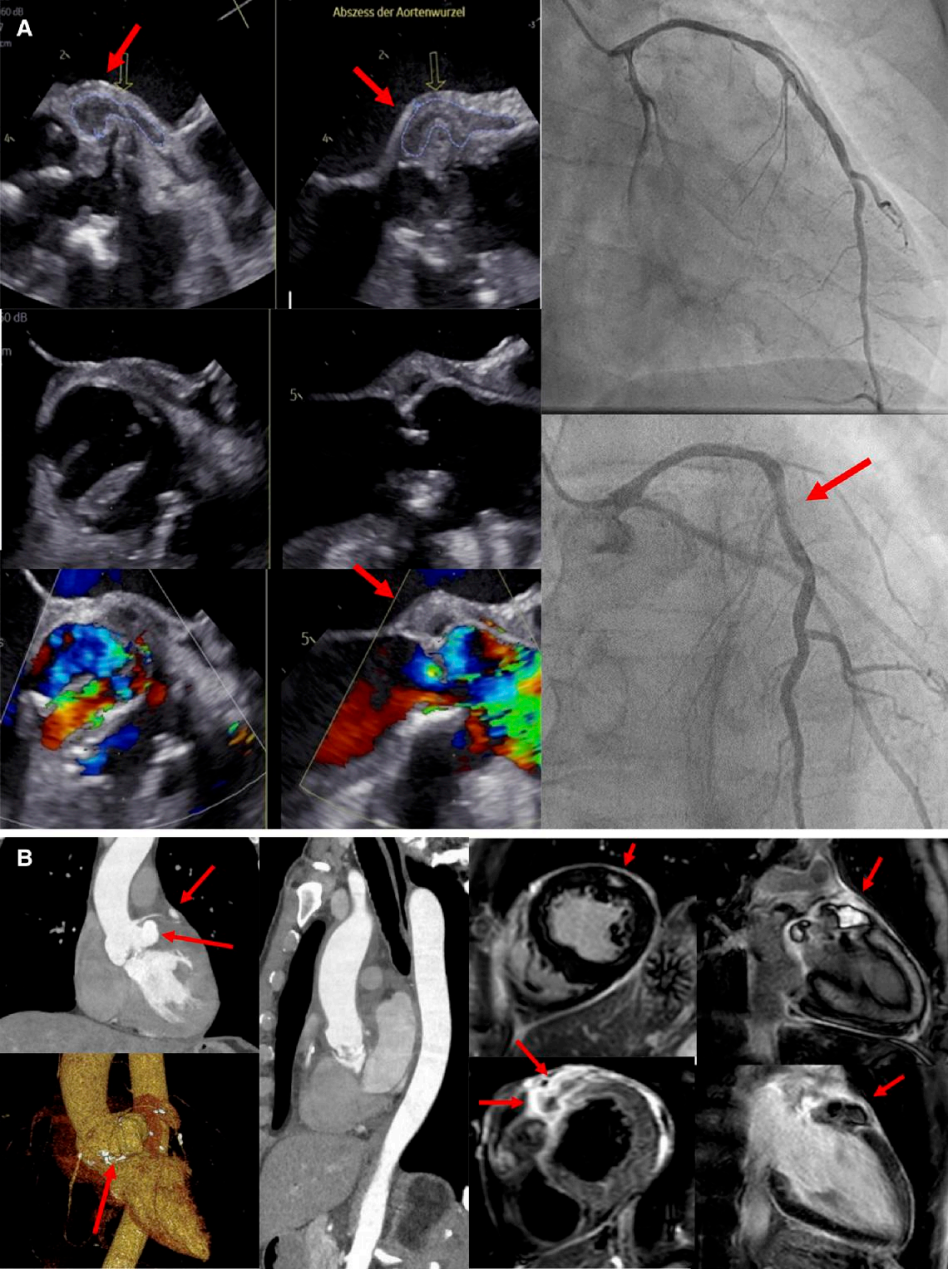
*(A)* Left side: Transoesophageal echocardiography demonstrating the aortic root abscess. On the right side: Coronary angiography showing normal left anterior descending (upper picture) and 60% stenosis of the circumflex artery (lower picture), most likely causing the, fake pectanginous symptoms due to the abscess additionally pressing on the artery. *(B)* Multimodal imaging methods presenting the massive aortic root abscess expanding from the anterior cusp of the bicuspid valve to the anterior basal ventricular myocardium. Left side: Computed tomography angiography demonstrating the large aortic root abscess with an axial size of ∼35 × 30 × 26 mm with expansion from the anterior cusp of the bicuspid aortic valve to the anterior basal ventricular myocardium. Right side, *top* row: Late gadolinium enhancement short-axis view showing another abscess formation at the end of the intramyocardial road of pus inside the lateral midventricular wall, firstly interpreted as microembolic lesion (short-axis view, second picture from the upper right). On the right side: a magnitude image for better visualization of the aortic root abscess (2-CH view). *Bottom* row: a T_2_ SA demonstrating myocardial oedema. Left side: Late gadolinium enhancement 2-CH view with the intramyocardial abscess.

Due to intermittently occurring chest pain episodes and elevated troponin levels, the decision was made to promptly proceed with invasive coronary angiography (ICA). Invasive coronary angiography revealed one borderline significant stenosis at the proximal site of the circumflex artery, while left anterior descending (LAD) and right coronary artery presented smooth vessel walls (*Figure 2A*).

Viral myocarditis was considered as a differential diagnosis, three sets of blood cultures were drawn, and a CMR was scheduled. Cardiac magnetic resonance imaging was done the following day and revealed a large vegetation at the bicuspid aortic valve and an extensive subvalvular ARA in the area of ​​both valvular pockets, expanding intramyocardially (*Figure 2B*). Immediate empiric antibiotic treatment for IE was initiated based on blood cultures detecting *gram-positive cocci*. After receiving the growth and sensitivity report of multiple blood cultures containing *S. epidermidis* sensitive to flucloxacillin, the treatment was adjusted accordingly.

The patient was referred for urgent cardiac surgery and completed pre-operative diagnostics. For precise surgical planning and capture of the course of the aorta, an additional CT angiography of the aorta was performed and confirmed the paravalvular abscess with expansion from the anterior cusp of the bicuspid valve to the anterior basal ventricular myocardium, forming several finger-shaped–like pus roads’ and a second hypodense abscess formation inside the lateral midventricular wall (*Figure 2A*).

The patient underwent aortic root and valve replacements by aortic homograft with reimplantation of the coronary arteries into the graft. Left anterior descending and circumflex artery were lengthened using the great saphenous vein. Furthermore, a saphenous magna vein bypass was sewn onto the marginal ramus, bypassing the circumflex stenosis. The anterior mitral valve leaflet got replaced, and the pulmonary artery was reconstructed. Post-operatively, the patient received intravenous flucloxacillin for 4 weeks, starting from the day of the first negative blood culture. He was then discharged to a rehabilitation facility with an additional 2-week course of oral antibiotics, consisting of rifampicin and levofloxacin, completing a total of 6 weeks of antibiotics. The first follow-up 1 month later, along with another follow-up at 8 weeks, revealed an excellent surgical outcome on CT imaging. The patient appeared well-recovered and physically fit and had already returned to work.

## Discussion

Infective endocarditis is a rare disease with an in-hospital mortality rate of 15–20%.^2^ One of the most severe, life-threatening complications of IE remains perivalvular and ARA formation due to the high risk of spontaneous rupture and continued spread of organisms within the aorta and surrounding organs.^3^ This complication presents with a notably high mortality rate ranging from 12 to 30% for native and up to 50% for prosthetic valve–associated ARA. Risk factors contributing to increased mortality include heart defects, prosthetic valves, a history of intravenous drug use, and delayed treatment initiation.^4^

This case highlights the importance of early diagnosis and high clinical suspicion of ARA. Aortic root abscess may present with a wide spectrum of clinical manifestations, often including unspecific symptoms or unusual ECG changes such as PR interval lengthening, higher degree atrioventricular (AV) block or ischaemic changes, complicating early diagnosis. In the setting of IE, ischaemic changes are often attributed to pre-existing CAD or embolism from a vegetation. Another explanation is occlusion of the LAD and/or circumflex due to extrinsic compression from a subvalvular (pseudo-)aneurysm arising from an ARA.^5^ Due to its close proximity to the aortic valve, the compression of the AV node by the subvalvular ARA leads to a fluctuating intracardiac conduction in this case. The expansion of the abscess surrounding the circumflex artery was retrospectively held responsible for the ischaemic changes in inferior leads.

Other signs and symptoms of ARA might include increased WBC count, elevated inflammatory markers, skin abnormalities, or embolic events despite ongoing treatment. Antibiotic coverage and timely surgical intervention are crucial to improve prognosis and reduce mortality.

The modified duke criteria are commonly used for IE diagnosis with diagnosis categories of definite IE (two major or one major and three minor), possible IE (one major and one minor or three minor), or rejected IE (*Table 1*). The 2023 Duke-International Society for Cardiovascular Infectious Diseases Criteria propose significant updates, incorporating new microbiology diagnostics and imaging techniques, including cardiac CT (CCT). Additionally, intraoperative inspection was added as part of the new Surgery Major Clinical Criterion^6^ (*Table 2*). Transoesophageal echocardiography remains the gold standard for ARA diagnosis due to superior anatomical definition, while TTE is the initial imaging modality of choice for the diagnosis of IE, but its sensitivity is examiner- and patient-dependent. Newer imaging modalities such as CCT or CMR are recommended when conventional echocardiography is inconclusive.^6,7^ The 2020 American College of Cardiology/American Heart Association for the management of patients with valvular heart disease recommended the use of CCT as a complementary imaging modality for IE, and the 2023 ESC guidelines for the management of IE ranked CCT even as a class 1b recommendation in confirming IE diagnosis in patients with possible native valve endocarditis.^1,6,8^ Additionally, patients with elevated hsT levels (>14 ng/L), and inconclusive ECG could be saved by an ICA by upfront imaging with CT of the coronary artery (CTCA) to rule out CAD in suspected CAD,^9^ which might also reduce the risk of germ dissemination from vegetations.

**Table 1:** **Definition of infective endocarditis according to the modified Duke criteria from 2023** ^6,10^

Definite infective endocarditis
**Pathological criteria** • Microorganisms: demonstrated by culture or histological examination of a vegetation, a vegetation that has embolized or an intracardiac abscess specimen; or of a cardiac tissue, of an explanted prosthetic valve or sewing ring, of an ascending aortic graft (with concomitant evidence of valve involvement), or of an endovascular intracardiac implantable electronic device • Pathological lesions: vegetation or intracardiac abscess confirmed by histologic examination showing active endocarditis Clinical criteria (see *Table 2* for specific definitions) • Two major criteria • One major criterion and three minor criteria • Five minor criteria
**Possible infective endocarditis**
• One major criterion and one minor criterion • Three minor criteria
**Rejected infective endocarditis**
• Firm alternate diagnosis • Resolution of symptoms suggesting endocarditis with antibiotic therapy for ≤4 days • No pathologic evidence of infective endocarditis at surgery or autopsy, with antibiotic therapy for ≤4 days; or • Does not meet criteria for possible infective endocarditis, as above

**Table 2:** **2023 updated definition of the terms used in the modified Duke criteria for the diagnosis of IE** ^6^

Criteria	Description
I. Major criteria	
A. Microbiologic major criteria	(1) Positive blood cultures: Microorganisms commonly causing IE isolated from ≥2 separate blood culture sets (typical) Microorganisms occasionally/rarely causing IE from ≥3 blood culture sets (non-typical).
	(2) Positive laboratory tests: PCR/nucleic acid–based technique for *Coxiella burnetii*, *Bartonella* species, or *Tropheryma whipplei* *C. burnetii* antiphase I IgG > 1:800d IFA for *Bartonella* spp. IgG ≥ 1:800d
B. Imaging major criteria	(1) Echocardiography and cardiac CT Echocardiography and/or cardiac CT showing vegetation, valvular/leaflet perforation, valvular/leaflet aneurysm, abscess, pseudoaneurysm, or intracardiac fistula Significant new valvular regurgitation on echocardiography as compared with previous imaging New partial dehiscence of prosthetic valve as compared with previous imaging (2) [18F]FDG PET/CT showing abnormal metabolic activity involving a native or prosthetic valve, ascending aortic graft, intracardiac device leads, or other prosthetic material.
C. Surgical major criteria	Evidence of IE documented by direct inspection during heart surgery. Surgery neither major imaging criteria nor subsequent histologic or microbiologic confirmation
II. Minor criteria	
A. Predisposition	Previous IE, prosthetic valve, valve repair, congenital heart disease, regurgitation/stenosis, endovascular cardiovascular implantable electronic device infection, hypertrophic obstructive cardiomyopathy, and injection drug use
B. Fever	Documented temperature > 38.0°C (100.4°F).
C. Vascular phenomena	Evidence of arterial emboli, abscesses, aneurysms, haemorrhages, Janeway lesions, and purpura
D. Immunologic phenomena	Positive rheumatoid factor, Osler’s nodes, Roth spots, immune complex–mediated glomerulonephritis
E. Microbiologic evidence	Positive blood cultures or nucleic acid tests not meeting major criteria; organism consistent with IE from a sterile site; skin bacterium by PCR on valve/wire
F. Imaging criteria	Abnormal [18F]FDG PET/CT within 3 months after prosthetic valve/graft/lead implantation.
G. Physical examination	New valvular regurgitation identified on auscultation if echocardiography is not available. Worsening/changing pre-existing murmur not sufficient

**Figure 3:**
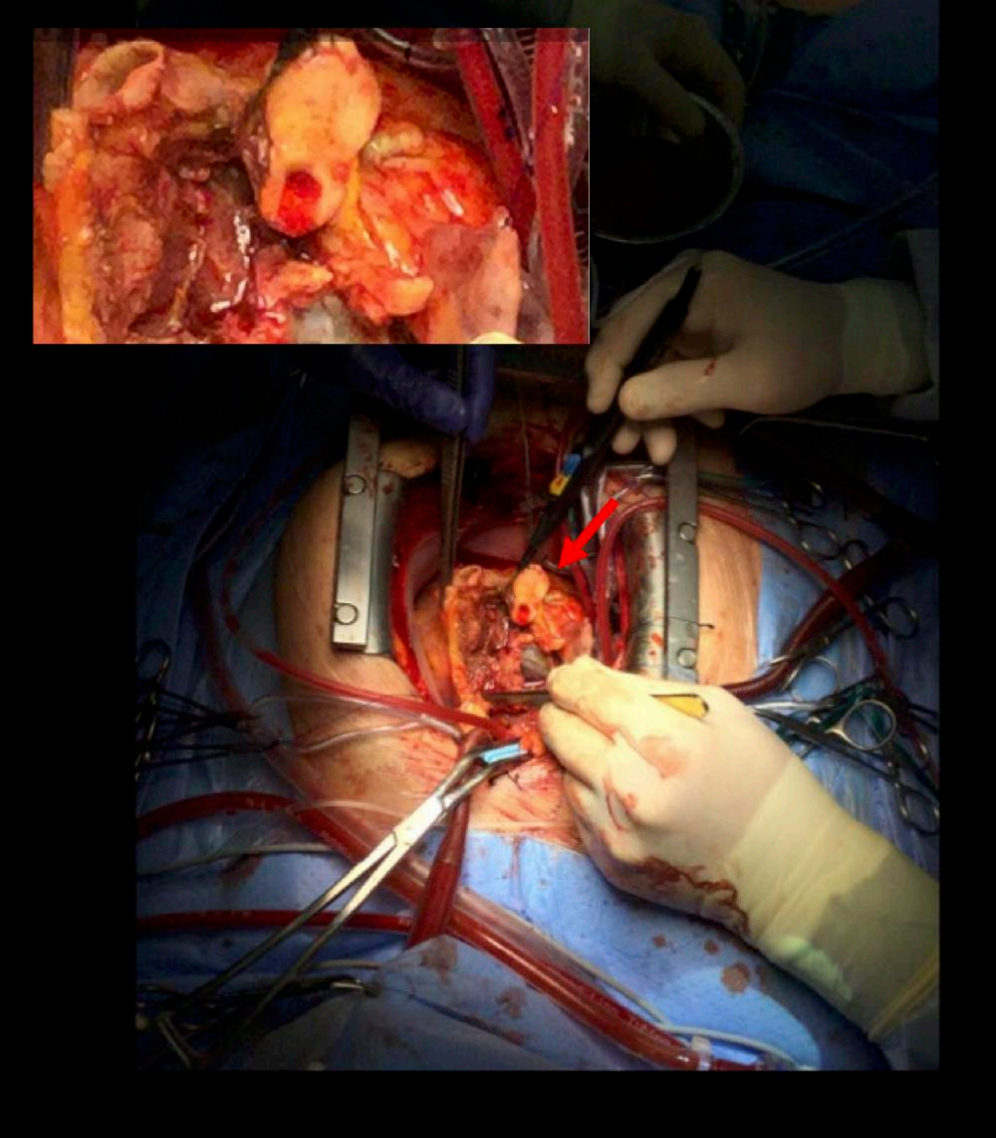
Open situs during cardiac surgery. The picture on the upper left demonstrating in more detail the aortic root abscess extending from the aortic valve to the left main truncus, forming a visible sheath of pus surrounding the circumflex artery.

In our case, IE was suspected after CMR, which was performed to rule out myocarditis. The CMR imaging detected the large aortic valve vegetation and giant ARA, forming intramyocardial roads of pus down the lateral wall, surrounding and as the surgical examination showed (*Figure 3*), most likely compressing the circumflex artery, which was possibly the reason for the pectanginous symptoms in this patient.^5^ Cardiac magnetic resonance imaging is well suited for diagnosing complications of IE. In comparison with echocardiography, CMR is more sensitive with regard to tissue alterations (e.g. abscess formation) due to its unique ability of assessing myocardial viability. Therefore, in case of suspected endocarditis, we recommend a prompt approach with asservation of blood cultures, initiation of empiric i.v. antibiotics, and performing readily available imaging like CCT, in addition to TEE and TTE. Cardiac CT offers the option of simultaneous visualization of the coronary arteries and valves and can aid to detect the cause in cases with additional ischaemic ECG changes. For complex lesions, in unclear or highly suspicious situations, we advocate to perform CMR due to its superior tissue delineation as an adjunct examination.^11^ Furthermore, we advise a rapid multidisciplinary approach and a joint discussion of surgical intervention if applicable.

## Conclusions

Challenges persist in diagnosing IE and ARA in atypical cases such as those with inconclusive or ischaemic ECG changes. This case highlights the importance of considering IE as an early differential and demonstrates the significance of CCT and CMR imaging, especially when gold standard echocardiography remains inconclusive. Recent guidelines underscore the role of CCT and CMR in diagnosing and managing IE emphasizing its value in identifying perivalvular complications and extra-cardiac manifestations. Cardiac CT used in suspected CAD can aid in minimizing unnecessary invasive procedures by evaluating coronary anatomy and vascular structure, shorten time to diagnosis, and help in pre-operative planning.

## Lead author biography


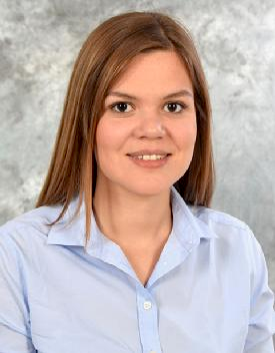


Stephanie Wissel received her medical degree from the First Faculty of Medicine at Charles University in Prague in 2019. She then began her residency in cardiology at the University Hospital in Würzburg, Germany, where she is currently still in training.

##  

**Consent:** We have obtained the patient’s informed consent for publication, following the best practice guidelines outlined by Committee on Publication Ethics.

**Funding:** None declared.

## Data Availability

The data underlying this work can be obtained upon reasonable request to the authors.
